# Connexin-Dependent Neuroglial Networking as a New Therapeutic Target

**DOI:** 10.3389/fncel.2017.00174

**Published:** 2017-06-26

**Authors:** Mathieu Charvériat, Christian C. Naus, Luc Leybaert, Juan C. Sáez, Christian Giaume

**Affiliations:** ^1^TheranexusLyon, France; ^2^Department of Cellular and Physiological Science, Life Science Institute, University of British ColumbiaVancouver, BC, Canada; ^3^Physiology Group, Department of Basic Medical Sciences, Faculty of Medicine and Health Sciences, Ghent UniversityGhent, Belgium; ^4^Departamento de Fisiología, Pontificia Universidad Católica de ChileSantiago, Chile; ^5^Centro Interdisciplinario de Neurociencias de Valparaíso, Instituto MilenioValparaíso, Chile; ^6^Center of Interdisciplinary Research in Biology, Collège de FranceParis, France

**Keywords:** astrocyte network, connexin, gap junction, neuroglial interaction, glia

## Abstract

Astrocytes and neurons dynamically interact during physiological processes, and it is now widely accepted that they are both organized in plastic and tightly regulated networks. Astrocytes are connected through connexin-based gap junction channels, with brain region specificities, and those networks modulate neuronal activities, such as those involved in sleep-wake cycle, cognitive, or sensory functions. Additionally, astrocyte domains have been involved in neurogenesis and neuronal differentiation during development; they participate in the “tripartite synapse” with both pre-synaptic and post-synaptic neurons by tuning down or up neuronal activities through the control of neuronal synaptic strength. Connexin-based hemichannels are also involved in those regulations of neuronal activities, however, this feature will not be considered in the present review. Furthermore, neuronal processes, transmitting electrical signals to chemical synapses, stringently control astroglial connexin expression, and channel functions. Long-range energy trafficking toward neurons through connexin-coupled astrocytes and plasticity of those networks are hence largely dependent on neuronal activity. Such reciprocal interactions between neurons and astrocyte networks involve neurotransmitters, cytokines, endogenous lipids, and peptides released by neurons but also other brain cell types, including microglial and endothelial cells. Over the past 10 years, knowledge about neuroglial interactions has widened and now includes effects of CNS-targeting drugs such as antidepressants, antipsychotics, psychostimulants, or sedatives drugs as potential modulators of connexin function and thus astrocyte networking activity. In physiological situations, neuroglial networking is consequently resulting from a two-way interaction between astrocyte gap junction-mediated networks and those made by neurons. As both cell types are modulated by CNS drugs we postulate that neuroglial networking may emerge as new therapeutic targets in neurological and psychiatric disorders.

## Introduction

In the eighteenth century, the neuron doctrine asserted that nerve tissues are composed of functional units called neurons. The concept of neuroglia, the connective tissue in which nervous system elements were embedded, proposed by Rudolf Virchow by the end of the last century (Virchow, 1859), has at least four different sub-populations of glial cells identified in the central nervous system (Kettenmann and Verkhratsky, 2008). Among them, astrocytes were considered as having a high level of dynamic and integrative interactions with neurons. When electrophysiological properties of astrocytes were discovered, neuroglial interactions were firstly theorized. The occurrence of electrotonic and dye coupling between those cells were demonstrated in the late 80s (Kettenmann and Ransom, 1988), followed by the description of the structures and the molecular nature of GJs that connect astrocytes (Giaume et al., 1991a; Nagy and Rash, 2000). In the 90s, communication between astrocytes via Ca^2+^ waves were identified, as well as tuning of neuronal activity, leading to the first documentation of bi-directional communication between astrocytes and neurons, and descriptions of “tripartite synapses” associating a pre-synaptic neuron, a post-synaptic neuron and an astrocyte (Araque et al., 1999; Hubbard and Binder, 2016). Interestingly, one astrocyte can be connected to up to 140,000 synapses (Bushong et al., 2002); astrocytes are organized in plastic and tightly regulated networks (Giaume and Liu, 2012). The role of astrocytes in complex functions is further emphasized by the glia-to-neuron ratio that increases with the evolution toward more and more adapted organisms (Verkhratsky and Nedergaard, 2016). This review focuses on how astrocytes and neurons communicate, encompassing the extent and the modulation of astrocyte networking. We will specifically emphasize the role of astrocyte connexins (Cxs), transmembrane proteins involved in cell-cell communication through GJs, as the role of astrocytes Cxs as hemichannels has been recently reviewed (Giaume et al., 2013; Orellana and Stehberg, 2014).

## Astrocyte networks are formed by GJs

### Astrocyte GJs are encoded by Cx30 and Cx43

Among the 11 Cxs detected in the rodent brain (Nagy et al., 2004), astrocytes express two major Cxs, Cx43, and Cx30, and possibly Cx26 (Perez Velazquez et al., 1996; Lynn et al., 2011; Wasseff and Scherer, 2011). In astrocytes, Cx43 is distributed in a plaque-like manner between cells and is also located elsewhere in these cells compatible with cytoplasmic pools or hemichannels. This Cx appears post-natally while Cx30 is expressed several weeks after birth (Kunzelmann et al., 1999). In primary cultures astrocytes express only Cx43 while Cx30 starts to be detected after 10 weeks, however, the addition of neurons induces expression of Cx30 in 3 week-old primary cultures (Koulakoff et al., 2008). Interestingly, Cx30/Cx30 and Cx43/Cx43 form functional homotypic channels, whereas heterotypic Cx30/Cx43 do not (Orthmann-Murphy et al., 2007). In general, Cx43 is predominant, but its expression varies by brain region and stage of development (Nagy et al., 2004). For instance, hypothalamic astrocytes express four times more Cx43 than striatal astrocytes, and are more highly coupled (Batter et al., 1992). White matter astrocytes express minimal or no Cx30 (Nagy et al., 1999). In contrast, in the thalamus Cx30 is more highly expressed than Cx43 (Griemsmann et al., 2015). Coupling between astrocytes is greatly reduced in cells lacking Cx43 and abolished when both Cx43 and Cx30 are absent (Perez Velazquez et al., 1996; Naus et al., 1997; Wallraff et al., 2006; Rouach et al., 2008; Roux et al., 2011), demonstrating that these two Cxs are the main molecular support for direct intercellular communication in astrocytes.

### Regulation of GJs in astrocytes

Neurotransmitters and second messenger pathways regulate gap junctional communication between astrocytes for review see Giaume et al. (2010). In cultured astrocytes “dye-coupling” can be increased by extracellular application of glutamate or high K^+^ solutions (Enkvist and McCarthy, 1994). Such K^+^-induced upregulation of gap junctional communication may involve the calmodulin kinase pathway (De Pina-Benabou et al., 2001). In the olfactory bulb (Roux et al., 2011) and the thalamus (Claus et al., 2016), gap junctional communication is controlled by neuronal activity and this regulation is due to Cx30 GJ channels (Roux et al., 2011). Glutamate or high K^+^ solution causes membrane depolarization and this might be linked to changes in coupling via changes in intracellular pH. Depolarization produces an alkaline shift in astrocytes that could enhance coupling (Ransom and Sontheimer, 1992). In addition, glutamate effects on gap junctional communication may contribute to neuronal activity-dependent control of Cx43 expression in astrocytes with important consequences for astrocyte coupling (Rouach et al., 2000). In contrast to the effect of neuronal activity, brain macrophages can reduce Cx43 expression in astrocytes (Rouach et al., 2002b; Faustmann et al., 2003). Other naturally occurring molecules, including endothelins (Blomstrand et al., 2004) and inflammatory cytokines (Duffy et al., 2000), have also been shown to downregulate Cx expression and function in astrocytes. These properties indicate that the extent of astroglial networks is under the control of a number of active compounds present in the brain, including neurotransmitters, which means that their size and probably their shape are plastic.

### Organization of astrocyte networks in anatomo-functional units

Interestingly, in brain areas characterized by a strong anatomo-functional organization, the network of coupled astrocytes follows such topographical organization. This is the case in layer IV of the somatosensory cortex, the so-called barrel cortex, where the distribution of Cx43 and Cx30 is enriched within the barrels compared to the inter-barrel space (Houades et al., 2008). This feature is also found for the intercellular communication since dye coupling experiments indicate that coupling is favored within the barrels, while astrocytes located in the septa are weakly coupled (Houades et al., 2008). Interestingly, such restricted networking has recently been found in the thalamus in the barreloid fields (Claus et al., 2016) that pertain to the same whisker sensory-motor pathway. Another study performed in the olfactory bulb has demonstrated that in the glomerular layer the expression of Cx43 and Cx30 is also increased in the glomeruli compared to the extraglomerular space and that the expression of the two Cxs is even segregated within the functional unit itself, Cx43 being more pronounced at the border of the glomeruli while Cx30 is rather detected into its center (Roux et al., 2011). These studies indicate that, at least in functional units, on the basis of Cx expression and channel function there is an overlap between neuronal circuitry and astroglial networking, suggesting strong correlations between local organization of astroglial networks and neuronal functions.

When considering glial networks two notions are important to take into account. First, not all astrocytes in a defined area are coupled together. This was reported in the hippocampus by performing dye coupling experiments in the eGFP-hGFAP mouse where it was observed that, in the coupling area defined by the intercellular spread of sulforhodamine B, several eGFP-positive astrocytes were excluded (Houades et al., 2006). Also in the olfactory bulb, olfactory ensheathing cells are reported to be preferentially coupled to a subset of their neighbors forming subdomains with the potential to affect specific axon fascicles (Rela and Szczupak, 2007). These observations suggest that astroglial networks, in addition to being plastic, do not involve all astrocytes of a considered location.

### Toward more complex networks

Additionally, there is now evidence for a panglial networking between two types of glial cells. This was already reported from co-culture studies that established gap junction (GJ)-mediated communication between astrocytes and oligodendrocytes (Kettenmann and Ransom, 1988) and that this heterotypic coupling requires differentiated oligodendrocytes (Venance et al., 1995; Niu et al., 2016). More recently, brain slice studies indicated that, depending on the brain area considered, this panglial networking was present in the neocortex, the hippocampus, and the thalamus (Griemsmann et al., 2015). Such heterotypic coupling may also have important consequences on the role of glial GJs as for instance recently reported for the intercellular trafficking of metabolic substrates between astrocytes and differentiated oligodendrocytes (Niu et al., 2016), thus adding further complexity in neuroglial networking interactions.

## Astrocyte networks modulate neuronal activities

As presented above and at least in specific brain areas, there is an overlap between neuronal circuitry and astroglial networking (Rela and Szczupak, 2007; Houades et al., 2008; Roux et al., 2011) or with the panglial networking (Claus et al., 2016). Such functional intersection can be correlated to the function of local astrocyte networks in complex cerebral functions, such as sleep-wake cycle regulation, and dysfunction (Franco-Perez et al., 2012), cognition (Dallerac and Rouach, 2016), behavior (Pannasch and Rouach, 2013), or sensory functions (Han et al., 2014).

### Roles of astrocyte networks as modulators of neuronal activities

Interestingly, astrocyte Cxs modulate adult neurogenesis in mice. Inactivation of Cx43 in astrocytes diminished neural progenitor proliferation and survival of new-born neurons, while ablation of Cx30 showed opposite effects (Liebmann et al., 2013). Moreover, it was shown that engrafting human astrocytes in recipient mice resulted in GJ-mediated coupling between host and engrafted astrocytes with a sharply enhanced long-term potentiation as well as their capacity for learning and memory (Han et al., 2013). More precisely, it was demonstrated that Cx43-mediated gap-junctional coupling between astrocytes is important in the neuron-glia interactions required for whisker-related sensory functions and plasticity. Indeed, mice with a knocked-down expression of Cx43 in astrocytes showed a reduction in their ability to sense the environment with their whiskers (Han et al., 2014).

Conversely, coupling-deficient astrocytes lead neurons to present a reduced threshold for the generation of epileptiform events (Wallraff et al., 2006). The role of astroglial networks for normal brain functions is also highlighted by impairments in sensorimotor and spatial memory tasks in double KO Cx43/Cx30 mice (Lutz et al., 2009), the modulation of exploratory and locomotor activity in Cx43 KO mice (Frisch et al., 2003; Theis et al., 2003) or the anxiogenic profile of Cx30 KO mice (Dere et al., 2003). Meanwhile, double KO Cx43/30 mice exhibit normal pain perception but do not present allodynia or hyperalgesia after spinal cord injury, contrary to simple KO Cx30 mice (Chen et al., 2012).

It was further shown by inactivating Cx43 and Cx30 that astroglial networks reduced synaptic transmission in hippocampal neurons (Pannasch et al., 2011). Additionally, GJ-mediated glial networks enable synaptic information transfer and limits the number of functional synapses, hence modulating the generation of neuronal network activities (Pannasch et al., 2012). In the end, astroglial networks were shown to modulate neuronal depolarization, release probability, and recruitment during bursting (Chever et al., 2016). Altogether, those experiments largely contribute to the hypothesis that Cx expression in astrocytes can contribute to modulate neuronal functions and synchrony.

### How do astrocyte networks tune neuronal functions?

**Figure 1:**
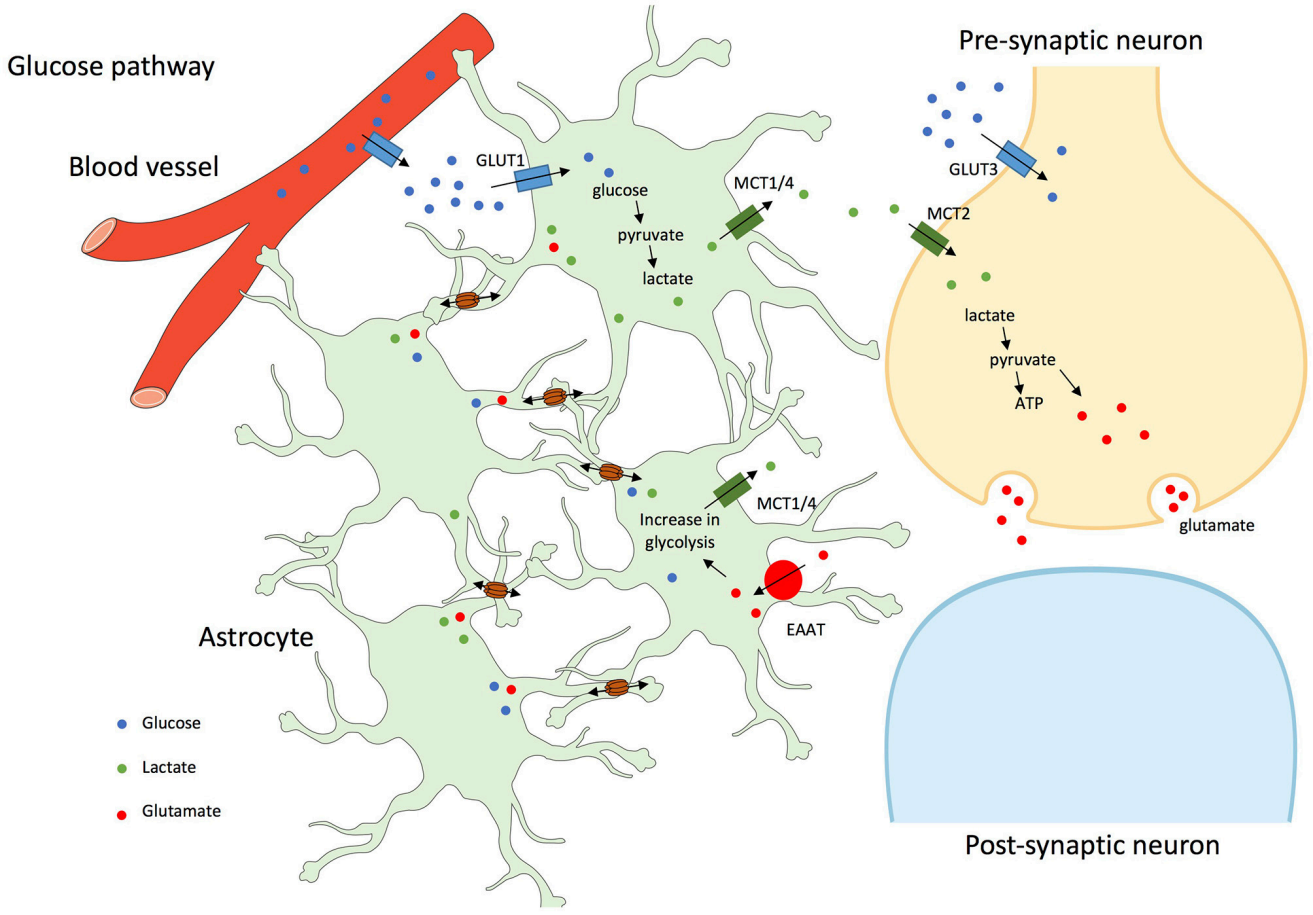
Glucose and glutamate pathways in astrocyte networks and interactions with neurons. In addition to a direct pathway to neurons by the extracellular space, glucose from blood vessels is taken-up through astrocytic end-feet or through GLUT1 (Glucose transporter 1) facilitated transport, and by neurons through GLUT3 (Glucose transporter 3) transport. In astrocytes, glucose is metabolized to lactate, which is exported through MCT1/4 (Mono-Carboxylate Transporter 1/4); lactate is shuffled into neurons by MCT2 (Mono-Carboxylate Transporter 2) and participates in the synthesis of glutamate, further stored in vesicles until exocytotic release. After neuronal firing, glutamate released into the synaptic cleft is removed by astrocytes through EAAT (Excitatory Amino Acid Transporter) and recycled as glutamine. This glutamate-glutamine cycling and the associated sodium entry consumes energy, which drives astrocytic glycolysis and results in lactate production (Pellerin et al., 2007). Glucose, lactate, and glutamate are transferred between connected astrocytes through GJs (Cx30 and Cx43; Leybaert, 2005; Orellana et al., 2009; Escartin and Rouach, 2013).

To test whether astrocyte networks contribute to glutamatergic synaptic activity, neuronal activity during exogenous glucose deprivation was monitored in mice. Lactate and glucose were demonstrated to be trafficked through astrocyte networks in the hippocampus and further used by neurons as an energetic substrate to sustain their excitatory activity. This directly suggests that astroglial networks are involved in energetic metabolite trafficking from astrocytes to neurons (Rouach et al., 2008; see Figure 1).

**Figure 2:**
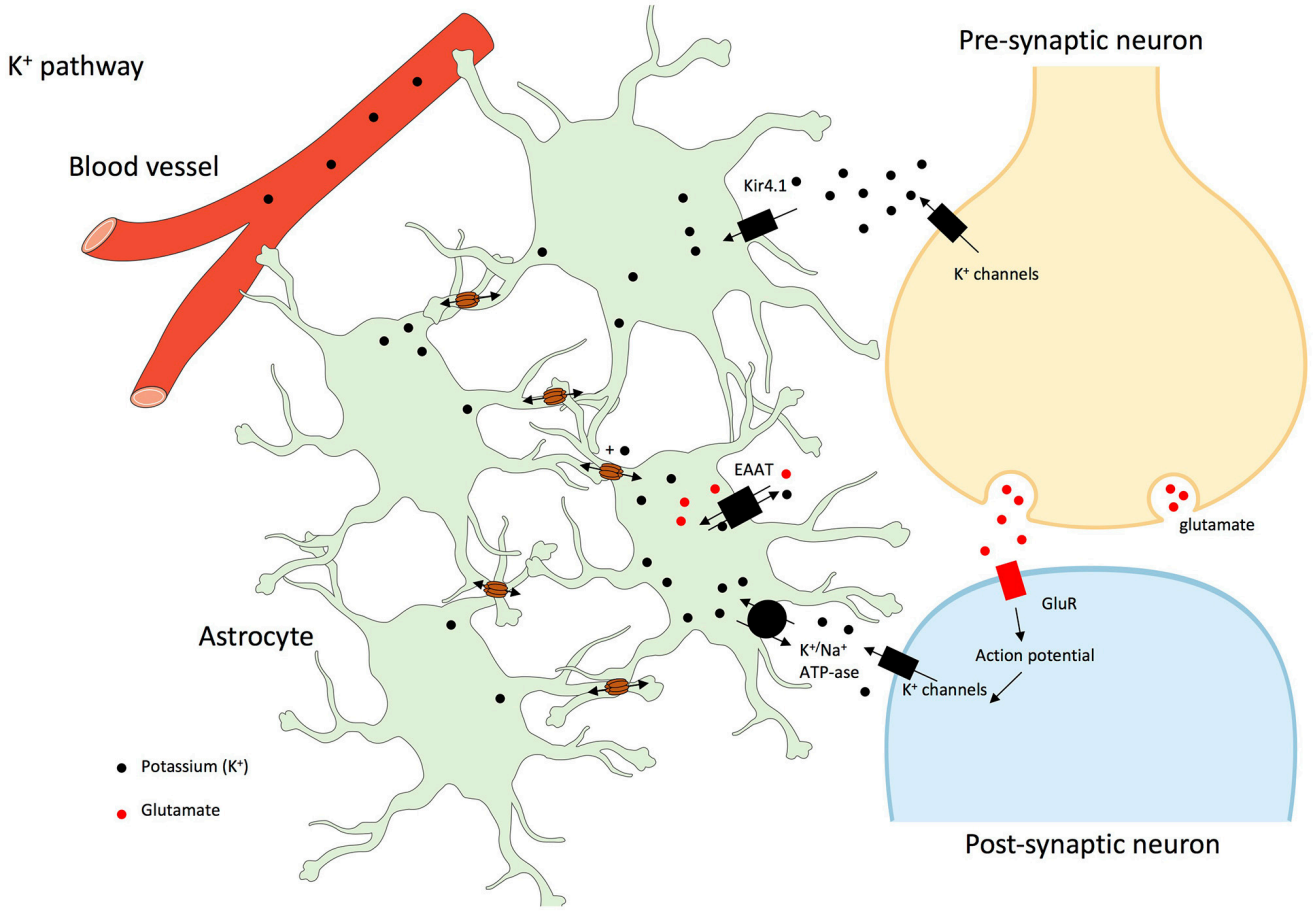
K^+^ pathway in astrocyte networks and interactions with neurons. Gap junctions play a central role in the dissipation of K^+^ and its spatial buffering by astrocytes. Indeed, glial networking disperses local extracellular K^+^ increases by transferring K^+^ from sites of elevated concentrations to sites of lower concentrations through gap junctions. Potassium ions are taken-up through Kir1.4 (Potassium inwardly-rectifying channel 1.4), EAAT or K^+^/Na^+^-ATP-ase (Rouach et al., 2000, 2004, 2008; Kofuji and Newman, 2004; Giaume et al., 2010; Pannasch and Rouach, 2013). In addition, at least in olfactory glomeruli, high external K^+^ concentrations increases Cx30-mediated intercellular coupling which facilitates K^+^ buffering by astroglial networks (Roux et al., 2011).

It has also been demonstrated that double Cx43-Cx30 KO mice have increased hippocampal synaptic transmission, and impaired long-term synaptic plasticity. Indeed, astrocyte GJs facilitate extracellular glutamate and potassium ion (K^+^) removal during neuronal activity (see Figure 2). Synaptic function is hence limited and controlled by astrocyte networks, through the impact on extracellular homeostasis notably on glutamate and K^+^ (Wallraff et al., 2006; Pannasch et al., 2011). Moreover, it was described that Cx30 controlled the setting of excitatory synaptic strength, justifying the role it plays in long-term synaptic plasticity and in hippocampus-based contextual memory (Pannasch et al., 2014).

Finally, Ca^2+^ signaling in astroglial networks can trigger glutamate release and hence neuron depolarization and firing (Kang et al., 2005), synchronize neuronal activities (Poskanzer and Yuste, 2011, 2016) and underlie hippocampal synaptic plasticity involved in learning and memory (Serrano et al., 2006). Additionally, in mice grafted with human astrocytes, intercellular Ca^2+^ waves were three-time faster than in normal conditions, and basal transmission and synaptic plasticity were enhanced, pointing the role of those waves in neuronal control (Han et al., 2014).

**Figure 3:**
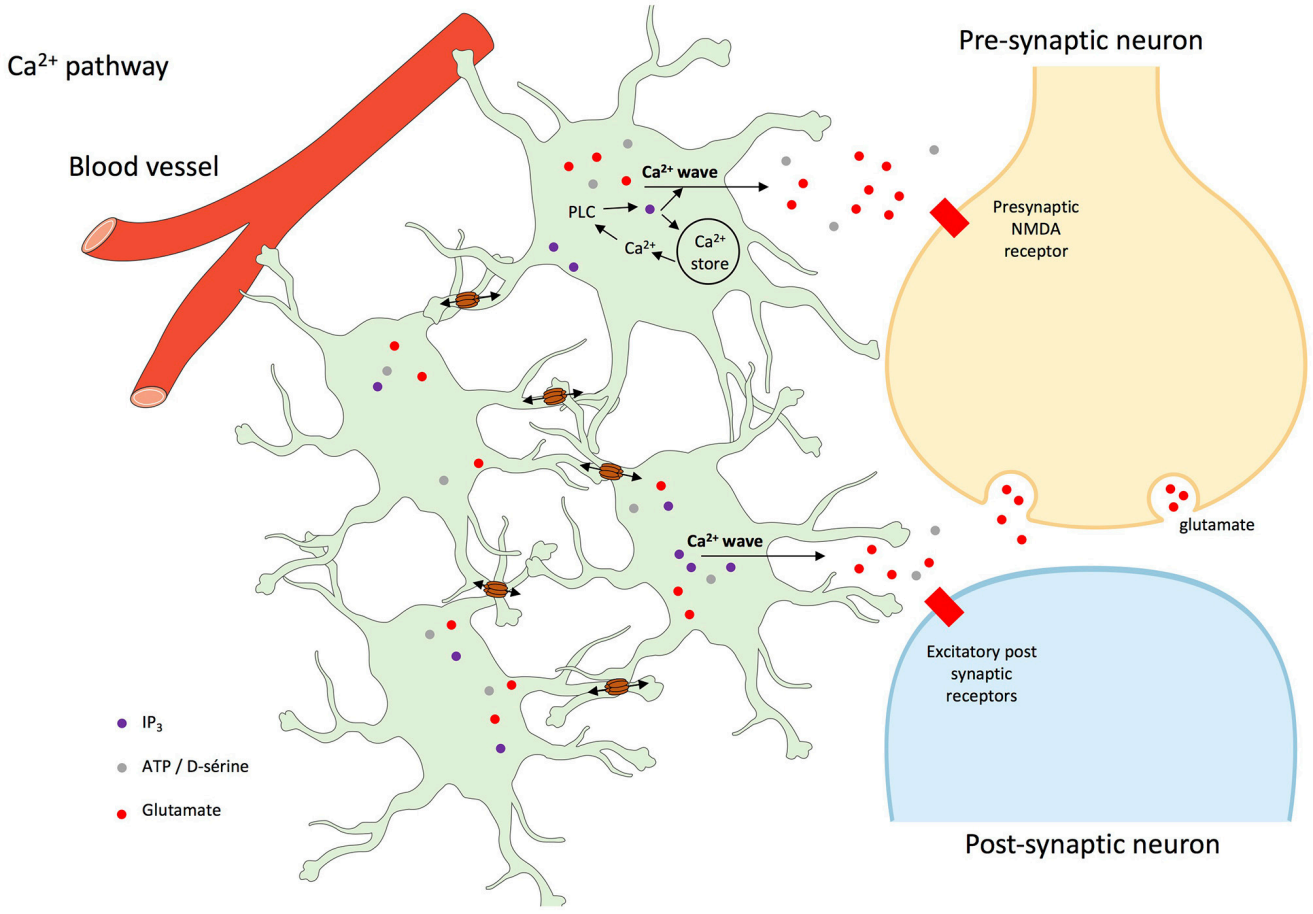
Intercellular Ca^2+^ waves in astrocyte networks and interactions with neurons. The rise of intracellular Ca^2+^ concentration in astrocytes usually involves the activation of G-protein-coupled receptors, activation of phospholipase C (PLC), and the production of inositol trisphosphate (IP_3_), which leads to Ca^2+^ release from the endoplasmic reticulum (ER). Elevation of cytoplasmic Ca^2+^ concentration further affects several plasma membrane proteins, such as metabotropic receptors, K^+^ and Ca^2+^ channels, Na^+^/Ca^2+^ exchanger, and Ca^2+^-ATPase. Once triggered, intracellular Ca^2+^ waves can be transmitted as intercellular Ca^2+^ waves to neighboring cells through the diffusion of inositol trisphosphate (IP_3_) production and Ca^2+^ through gap junctions, and subsequent release of Ca^2+^ from the endoplasmic reticulum (Cotrina et al., 1998; Leybaert et al., 1998; Scemes et al., 1998; Scemes and Giaume, 2006; Leybaert and Sanderson, 2012; De Bock et al., 2014). Ca^2+^-dependent release of gliotransmitters then target neuronal receptor but also astroglial receptors that participate to and amplify the process of propagation of intercellular Ca^2+^ waves (Scemes and Giaume, 2006).

While in the hippocampus Cx30 and Cx43 each contribute to approximately half of the astroglial communication (Rouach et al., 2008), they play different roles in terms of neuronal modulation (Dere et al., 2003; Frisch et al., 2003; Theis et al., 2003; Lutz et al., 2009; Roux et al., 2011; Chen et al., 2012), as recently reviewed (Pannasch and Rouach, 2013). Nevertheless, data are in favor of a role of astroglial network control of neurons through at least spatial K^+^ buffering, glutamate clearance, and energy metabolite trafficking, potentially mediated by the spreading of astrocyte activation by cell-to-cell propagating intercellular Ca^2+^ waves (see Figure 3).

## Neuronal activity regulates astrocyte networks

Neuronal activity is modulated by astrocyte networks as described above, but the opposite, i.e., a tight control of astroglial Cxs by neurons, has also been amply documented the literature (Rouach et al., 2004; Giaume et al., 2010).

### Evidence for regulation of astroglial networks by neurons

Neurotransmitters can either increase or decrease the strength of GJ coupling depending on the receptor subtypes that are involved (Giaume and McCarthy, 1996), for instance noradrenaline (Giaume et al., 1991b), glutamate (Enkvist and McCarthy, 1994; Muller et al., 1996), NMDA/acetylcholine (Rouach et al., 2002c), or serotonin (Moore and Burt, 1995) modulate Cx43 gap junctional communication (GJC).

There is strong evidence that neurons upregulate GJC. Indeed, experimental work has indicated that in co-cultures the presence of neurons largely enhanced astroglial coupling via Cxs (Rouach et al., 2000; Koulakoff et al., 2003, 2008). This effect was attributed to astrocyte differentiation by diffusible factors from neurons (Fischer and Kettenmann, 1985). Cellular coupling enhancement depends on the age and density of neurons, as neuronal maturation is required to observe the increase (Rozental et al., 1998; Rouach et al., 2000). Similarly, neuronal injury leads to an increase of GJC in co-cultured astrocytes (Anders and Woolery, 1992) or in astrocytes *in situ* (Rohlmann et al., 1993; Hanani et al., 2002). Additionally, nerve stimulation increases gap junctional communication (Marrero and Orkand, 1996) and dephosphorylates Cx43 in astrocytes (Li and Nagy, 2000).

On the opposite, the stimulatory effect of neurons on astroglial GJC was prevented in several conditions in which neuronal functions were altered, as after treatment with TTX, used to inhibit neuronal spontaneous activity (Muller et al., 1996; Rouach et al., 2008; Serrano et al., 2008; Roux et al., 2011). For instance, distant TTX infusion in Schäffer collaterals reduces the diffusion of a fluorescent glucose analog through astrocyte gap junctions in the stratum oriens by up to 35%, while the diffusion of passive dyes is not affected (Rouach et al., 2008). However, in the thalamus where Cx30 expression prevails in astrocytes, TTX reduces gap junction permeability for both types of molecules (Claus et al., 2016). In addition in the olfactory bulb where Cx30 also prevails, TTX or sensory deprivation also blocks plasticity of astrocyte networks in the rodent olfactory glomeruli (Roux et al., 2011).

### Cx30 and Cx43, major determinants of astrocyte network plasticity induced by neuronal activity

In the hippocampus, the regulation of astroglial networking by neurons is not observed with passive fluorescent dyes, indicating that neuronal activity does not directly regulate GJ permeability but rather triggers neuronal energetic demand through astrocyte gap junctions (Rouach et al., 2008). As indicated above, astrocytes are mainly coupled with Cx30- and Cx43-forming junctional plaques (Rouach et al., 2002a, 2004; Koulakoff et al., 2008). Interestingly, in cell cultures, neurons up-regulate the expression of Cx43; they also induce Cx30 expression in astrocytes located in proximity to neuronal somata. In Cx43-lacking astrocytes, the induction of Cx30 by neuronal presence allows the restoration of functional gap junctional communication mediated by Cx30 (Koulakoff et al., 2008). Additionally, in olfactory bulb acute slices, modulation of GJC by neuronal activity is still present in Cx43 KO but not in Cx30 KO mice. This indicates that in glomerular areas (i) Cx43-formed channels are insensitive to neuronal activity, and (ii) Cx30-formed channels are the molecular target of neuron-dependent modulation of astrocyte-astrocyte communication (Roux et al., 2011). Interestingly, a recent study indicates that in the thalamus dye coupling in glial networks is also dependent on neuronal activity (Claus et al., 2016). In this structure Cx30 is also predominant (Griemsmann et al., 2015), as in the olfactory bulb, which reinforces the idea that the regulation of this Cx is the target for the neuronal control of glial networks.

Several mechanisms have been proposed to mediate neuronal induced up-regulation of gap junctional communication. Depolarization mediated by activity-dependent K^+^ release has been demonstrated to enhance GJ coupling in astrocytes (Enkvist and McCarthy, 1994; Roux et al., 2011), with the participation of CAMKII-dependent phosphorylation of Cx43 (De Pina-Benabou et al., 2001). Glutamate is also a mediator of this modulation, however with confounding results depending on the involved receptor subtypes (Giaume, 2010; Giaume et al., 2010; Escartin and Rouach, 2013).

### Why are astrocyte networks plastic upon neuronal activity?

Hence, continuous interactions between neurons and astrocytes are required to maintain high levels of Cx expression and function in astrocytes (Koulakoff et al., 2008; Rouach et al., 2008). Collectively, these data indicate that the size of the astroglial metabolic network is enlarged as overall neuronal activity increases, and reduced while neuronal activity diminishes or is abolished (see Table 1).

**Table 1:** Evidence of modulation of astroglial connexin by neurons.

**Model**	**Effector**	**Effect of neurons on astrocyte Cx**		**Technique used**	**Comments**	**References**
		**Cx expression**	**GJ function**			
Cerebellar co-cultures	Neuronal co-culture	ND	↑	Intracellular injections of LY	Effect still present without physical contact	Fischer and Kettenmann, 1985
Whole brain co-cultures	Neuronal death	ND	↑	Laser-injury of neurons, gapFRAP	Increase in GJ after neuronal death, without physical contact	Anders and Woolery, 1992
Rat facial nucleus	Nerve lesion	↑ Cx43	ND	IHC	Increase of Cx43 expression after facial nerve lesion	Rohlmann et al., 1993
Frog optic nerve	Nerve stimulation	ND	↑	Intracellular injections of LY, IHC	Increase of dye-coupling following nerve stimulation	Marrero and Orkand, 1996
Rat spinal cord astrocyte cultures	Nerve stimulation	Cx43 dephosph.	ND	IHC	Dephosphorylation observed after sciatic nerve stimulation	Li and Nagy, 2000
Rat striatal co-cultures	Neuronal co-culture	↑ Cx43	↑	LY injection, immunoblotting, calcium imaging	Activity-dependent up-regulation	Rouach et al., 2000
Rat brain	Kainic-induced seizures	↑ Cx30	ND	PCR, IHC	Cx30 mRNA and protein increased after seizures	Condorelli et al., 2002
Mouse dorsal root ganglia	Nerve injury	ND	↑	Intracellular injection of LY, electron microscopy	Increase in dye coupling and density of GJ after axotomy	Hanani et al., 2002
Mouse cortical co-cultures	Neuronal co-culture	↑ Cx43Cx30	↑	Scrape-loading, immunoblotting, immunofluorescence	Increase in Cx30 GJC from Cx43 KO mice	Koulakoff et al., 2003
Mouse neuron-astrocyte co-cultures	Neurons	↑ Cx43↑ Cx30	↑	WB, IHC, scrape-loading	Increase of dye-coupling with neurons, reduction of dye-coupling after neuronal depletion (NMDA-excitotoxicity)	Koulakoff et al., 2008
	Neurons + NMDA	↓ Cx43	↓			
		↓ Cx30				
Mouse cortex	Excitotoxicity by kainic acid	↓ Cx43↓ Cx30	↓		Reduction of dye-coupling after neuronal depletion (kainate)	
Mouse hippocampus	TTX	ND	↓	Probing glucose trafficking with 2-NBDG and 6-NBDG	Glucose trafficking through GJ depending on neuronal activity	Rouach et al., 2008
	Picrotoxin	ND	↑			
	Evoked activity	ND	↑			
hGFAP-eGFP mouse hippocampus	TTX + NMDA application	ND	↓	Intracellular injection of biocytin	Reduction of dye-coupling after TTX treatment	Serrano et al., 2008
Mouse olfactory glomeruli	TTX or sensory deprivation	ND	↓	Intracellular injection of sulfo-rhodamine B	Reduction of dye-coupling after TTX treatment or sensory deprivation	Roux et al., 2011

*↑,increase; ↓,decrease*.

Astrocytes take-up glucose from blood flow, and glucose can be stored as glycogen and/or metabolized into lactate that can be released to neurons to fuel their tricarboxylic acid cycle. They provide local supply of energy substrates to neuronal surroundings (Magistretti, 2006; Pellerin et al., 2007; Belanger et al., 2011). Astrocytes are organized in plastic networks: those networks are confined to specific places as described in glomerular structures, where intraglomerular astrocytes are mainly coupled within the limits of a single glomerulus (Roux et al., 2011) within rodent barrels in the somatosensory cortex (Houades et al., 2006) and the barreloids in the thalamus (Claus et al., 2016). Such confinement potentially favors specific neuron-astrocyte interactions at a network level in physiological processes. As presented above, astrocyte networks are tightly regulated and gated by neuronal activity. Concerning the metabolic networking, this regulation involves glutamatergic signaling by AMPA receptors. Thus, it can be inferred that plasticity of the astroglial networking may result from an adaptation to neuronal demand, to reach more efficiently and distally the sites of high neuronal demand. Such a hypothesis has notably been established in specific areas of the hippocampus, in which neuronal activity changes the shape and extent of astroglial networks (Rouach et al., 2008).

## Astroglial Cxs and brain pharmacological agents

Drugs used to treat neurological and psychiatric disorders are generally studied through their neuronal effects. It is now clear that astrocytes are also targeted by those drugs, especially through the modulation of Cx30 and Cx43 levels of expression and function, those modulations are either neuron-dependent or not dependent upon neuronal activities (see Table 2), and might be linked to the aforementioned reciprocal interactions between neurons and astrocyte networks.

**Table 2:** Modulation of astroglial connexin by CNS pharmacological agents.

**Therapeutic class**	**Drug**	**Model**	**Dose**	**Cx expression**	**GJ function**	**HC function**	**References**
Tricyclic antidepressants	Amitriptyline	Primary cultured rat cortical astrocytes	48 μM, 48 h	↑ (mRNA, protein)	↑	ND	Morioka et al., 2014
		Primary cultured rat cortical astrocytes	10 μM, 24 h	→ (protein)	↓	↓	Jeanson et al., 2016a
		Primary cultured mouse cortical astrocytes	20 μM, 24 h	→ (protein)	↓	↓	Jeanson et al., 2016b
	Imipramine	Primary cultured mouse cortical astrocytes	20 μM, 24 h	→ (protein)	→	↓	Jeanson et al., 2016b
	Clomipramine	Primary cultured rat cortical astrocytes	10 μM, 48 h	↑ (protein)	ND	ND	Morioka et al., 2014
SSRI	Fluoxetine	Primary cultured mouse cortical astrocytes	10 μM, 24 h	→ (protein)	↓	↓	Jeanson et al., 2016b
		Rat prefrontal cortex	20 mg/kg/day, 21 days	↑ (protein)	ND	ND	Fatemi et al., 2008b
		Prefrontal cortex of chronically-stressed rats	10 mg/kg/day, 21 days	↑ (mRNA, protein)	↑	ND	Sun et al., 2012
		Hippocampus from corticosterone-treated rats	18 mg/kg, 1 month	↓ (protein)	ND	ND	Quesseveur et al., 2015
		Human astrocytoma cell lines	30–60 μM	↑ (mRNA, protein)	ND	ND	Mostafavi et al., 2014
	Paroxetine	Primary cultured mouse cortical astrocytes	5 μM, 24 h	→ (protein)	↑	↓	Jeanson et al., 2016b
	Fluvoxamine	Primary cultured rat cortical astrocytes	10 μM, 48 h	↑ (protein)	ND	ND	Morioka et al., 2014
NRI	Reboxetine	Primary cultured mouse cortical astrocytes	10 μM, 24 h	→ (protein)	→	↓	Jeanson et al., 2016b
SNRI	Duloxetine	Primary cultured mouse cortical astrocytes	5 μM, 24 h	→ (protein)	→	↓	Jeanson et al., 2016b
		Prefrontal cortex of chronically-stressed rats	10 mg/kg/day 21 days	↑ (mRNA, protein)	↑	ND	Sun et al., 2012
	Venlafaxine	Primary cultured mouse cortical astrocytes	5 μM, 24 h	→ (protein)	↓	↓	Jeanson et al., 2016b
Antipsychotics	Haloperidol	Rat prefrontal cortex	1.5 mg/kg/day 21 days	↓ (protein)	ND	ND	Fatemi et al., 2008b
	Lithium	Rat prefrontal cortex	1.5 mg/kg/day, 21 days	↓ (protein)	ND	ND	Fatemi et al., 2008b
	Clozapine	Rat prefrontal cortex	20 mg/kg/day, 21 days	↑ (protein)	ND	ND	Fatemi et al., 2008b
	Chlorpromazine	Primary cultured rat cortical astrocytes	25–100 μM, 1–24 h	↓ (protein)	↓	ND	Orellana et al., 2006
Psychostimulants	Modafinil	Mouse cortical slice (effects on astrocytes)	50–200 μM, 2 h	↑ (mRNA, protein)	↑	ND	Liu et al., 2013; Duchêne et al., 2016
Anti-epileptics	Levetiracetam	Primary cultured rat astrocytes + microglia	400 μM	↑ (protein)	↑	ND	Haghikia et al., 2008
	Valproic acid	Primary cultured rat astrocytes + microglia	300 μM	→ (protein)	ND	ND	Dambach et al., 2014
	Carbamazepine	Primary cultured rat astrocytes + microglia	40–400 μM	→ (protein)	ND	ND	Dambach et al., 2014
	Phenytoin	Primary cultured rat astrocytes + microglia	40–400 μM	→ (protein)	ND	ND	Dambach et al., 2014
	Gabapentin	Primary cultured rat astrocytes + microglia	50–500 μM	→ (protein)	ND	ND	Dambach et al., 2014
Anesthetics	Sodium oxybate	Mouse cortical slice (effects on astrocytes)	1 mM	ND	↓	ND	Liu et al., 2013
	Propofol	Mouse cortical slice	25–150 μM	ND	↓	↓	Liu et al., 2016
		Primary cultured mouse striatal astrocytes	400 μM	ND	↓	ND	Mantz et al., 1993
	Ketamine	Mouse cortical slice	50–300 μM	ND	↓	↓	Liu et al., 2016
	Dexmedetomidine	Mouse cortical slice	10 μM	ND	↓	→	Liu et al., 2016
	Halothane	Cx43 expressing cells	1–4 mM	ND	↓	ND	He and Burt, 2000
		Primary cultured rat cortical/striatal astrocytes	2 mM	ND	↓	ND	Dermietzel et al., 1991
		Primary cultured mouse striatal astrocytes	100 μM	ND	↓	ND	Mantz et al., 1993
	Enflurane	Primary cultured mouse striatal astrocytes	1.6 mM	ND	↓	ND	Mantz et al., 1993
	Isoflurane	Primary cultured mouse striatal astrocytes	1 mM	ND	↓	ND	Mantz et al., 1993

*↑,increase; ↓,decrease*.

### Diverse effects of antidepressants on astroglial Cxs

Amitriptyline, a tricyclic antidepressant widely used in neuropathic pain (Finnerup et al., 2015), significantly up-regulates Cx43 mRNA, protein and GJ channel function in astrocyte cultures. This up-regulation is not related to monoamines as noradrenaline, serotonin, and dopamine did not induce Cx43 expression. It is suggested that this mechanism depends on Cx43 phosphorylation through an increase of p38 MAPK activity by amitriptyline (Morioka et al., 2014). These results have been recently challenged (Jeanson et al., 2016a,b), as Cx43 GJs were inhibited (instead of promoted) by lower doses of amitriptyline than those used by Morioka et al. (2014). Possibly microglial cells may contribute to these distinct responses of GJ coupling to different doses of amitriptyline. Indeed, the number and activation status of microglial cells has been reported to interfere with Cx43 expression and function in astrocytes (Faustmann et al., 2003; Rouach et al., 2004; Même et al., 2006; Retamal et al., 2007).

Additionally, other studies have pointed out how tricyclic antidepressants such as clomipramine and imipramine modulate astrocyte Cx expression or functions (Morioka et al., 2014; Jeanson et al., 2016b). Of particular interest is the finding that Cx expression in astrocytes is modulated in animal models of depression as well as in depressed patients (Miguel-Hidalgo et al., 2014; Quesseveur et al., 2015; Nagy et al., 2017). Using relevant *in vivo* models (Hamon et al., 2008; M'Dahoma et al., 2015), it was demonstrated that mefloquine—at doses which do not affect neuropathic pain symptoms—significantly enhances the effects of amitriptyline on neuropathic pain (Jeanson et al., 2016a). Those data suggest a central role of astroglial Cxs as modulators of the pharmacological profile of antidepressants.

The analysis of others classes of antidepressants such as SNRI (Serotonin-Norepinephrine Reuptake Inhibitor), NRI (Norepinephrine Reuptake Inhibitor), and SSRI (Selective Serotonin Reuptake Inhibitor) has shown contrasting effects after treatment of astrocyte cultures (Sun et al., 2012; Morioka et al., 2014; Mostafavi et al., 2014; Jeanson et al., 2016b). Interesting data have been gathered from antidepressant-treated rodents: chronic treatment with fluoxetine (SSRI) increased Cx43 expression in rat prefrontal cortex (Fatemi et al., 2008b). Consistently, it has been shown that stressed rats presented a lower level of Cx43 expression and function in the cortex, while fluoxetine reversed those reductions (Sun et al., 2012). Alternatively, it has been proposed that antidepressants such as fluoxetine may exert their therapeutic activity by decreasing the expression and/or activity of Cx43 in astrocytes, resulting from a lower level of phosphorylation, in the hippocampus (Quesseveur et al., 2015). Those results emphasize the complex effects of different therapeutic classes of antidepressants on astroglial Cxs, with various outcomes on expression and GJ and hemichannel functions as well as regional properties.

### Astroglial Cxs and wake- or sleep-promoting drugs

The effects of numerous anesthetics on astroglial Cxs have been described for more than 20 years, notably in primary cultured astrocytes from the striatum or the cortex (Dermietzel et al., 1991; Mantz et al., 1993) or in astrocytes from cortical slices (Liu et al., 2013, 2016). Anaesthetics, either modulating GABA_A_ [halothane (Dermietzel et al., 1991; He and Burt, 2000), enflurane, isoflurane, or propofol (Mantz et al., 1993)], GABA_B_ receptors [sodium oxybate (Liu et al., 2013)], or NMDA (ketamine; Liu et al., 2016) or α2-adrenergic receptors (dexmedetomidine; Liu et al., 2016), show similar profiles, as they reduced astroglial GJ. Conversely, modafinil, a wake-promoting drug widely used in narcolepsy (Barateau et al., 2016) with a complex mechanism of action (Minzenberg and Carter, 2008), enhances astroglial Cx and more precisely Cx30 expression and GJ function (Liu et al., 2013). Additionally, when astrocyte Cxs are inhibited by a new blocker, flecainide (Picoli et al., 2012), the wake-promoting and pro-cognitive activity of modafinil is largely enhanced in rodent models. This effect likely depends on the reversion by flecainide of Cx30-dependent modafinil up-regulation of gap junctional coupling in astroglial networks. Surprisingly, GJ modulation by flecainide also modulates effects of modafinil on cataplexy, a pathognomonic symptom of narcolepsy, underlining once again the central role of astroglial Cx in complex behaviors (Duchêne et al., 2016).

Altogether, those data reinforce the role that astrocytes play as controllers of sleep rhythm (Fellin et al., 2012) and complex cognitive processes such as sleep (Franco-Perez et al., 2012), through the modulation of their network organization (Petit and Magistretti, 2016).

### Confounding effects of antipsychotics and anti-epileptics

Psychiatric disorders such as schizophrenia, bipolar disorders, or autism are characterized by marked signs of glial dysfunction (Yamamuro et al., 2015). More precisely, in autistic patients astroglial Cx43 expression is increased in superior frontal cortex (Fatemi et al., 2008a). Antipsychotic drugs have been developed since the 50s to reduce the symptoms of those disorders, and among them haloperidol, lithium, clozapine, or chlorpromazine. Those drugs generally reduce the expression of astrocyte Cxs (Orellana et al., 2006; Fatemi et al., 2008b). Concerning chlorpromazine, kinetic studies indicate that this reduction is achieved through an indirect mechanism involving a complex signaling cascade from drug application to effects on GJ channels, including modulation by alteredCx43 phosphorylation. Alternatively, a cellular redistribution of GJs by chlorpromazine has also been suggested to be at the origin of the reduction of Cx43 levels (Orellana et al., 2006). Astrocyte Cxs are also involved in the pathophysiology of epilepsy (Steinhauser et al., 2016), as they are often up-regulated in this pathology. In line with this, their blockade is considered to act in an anticonvulsant manner; however, opposite (pro-convulsing) effects have also been reported (Carlen, 2012). Anti-epileptic drugs (valproic acid, carbamazepine, phenytoin, or gabapentin) generally do not modulate Cx expression (Dambach et al., 2014). However, levetiracetam enhances the expression of Cx43 and function of GJ in astrocyte-microglia mixed cultures, allegedly through an anti-inflammatory process (Haghikia et al., 2008). Finally, the effect of anti-epileptic drugs are also known to have an effect on neuronal Cxs (Mylvaganam et al., 2014) but this is beyond the scope of the present review.

## Conclusion

**Figure 4:**
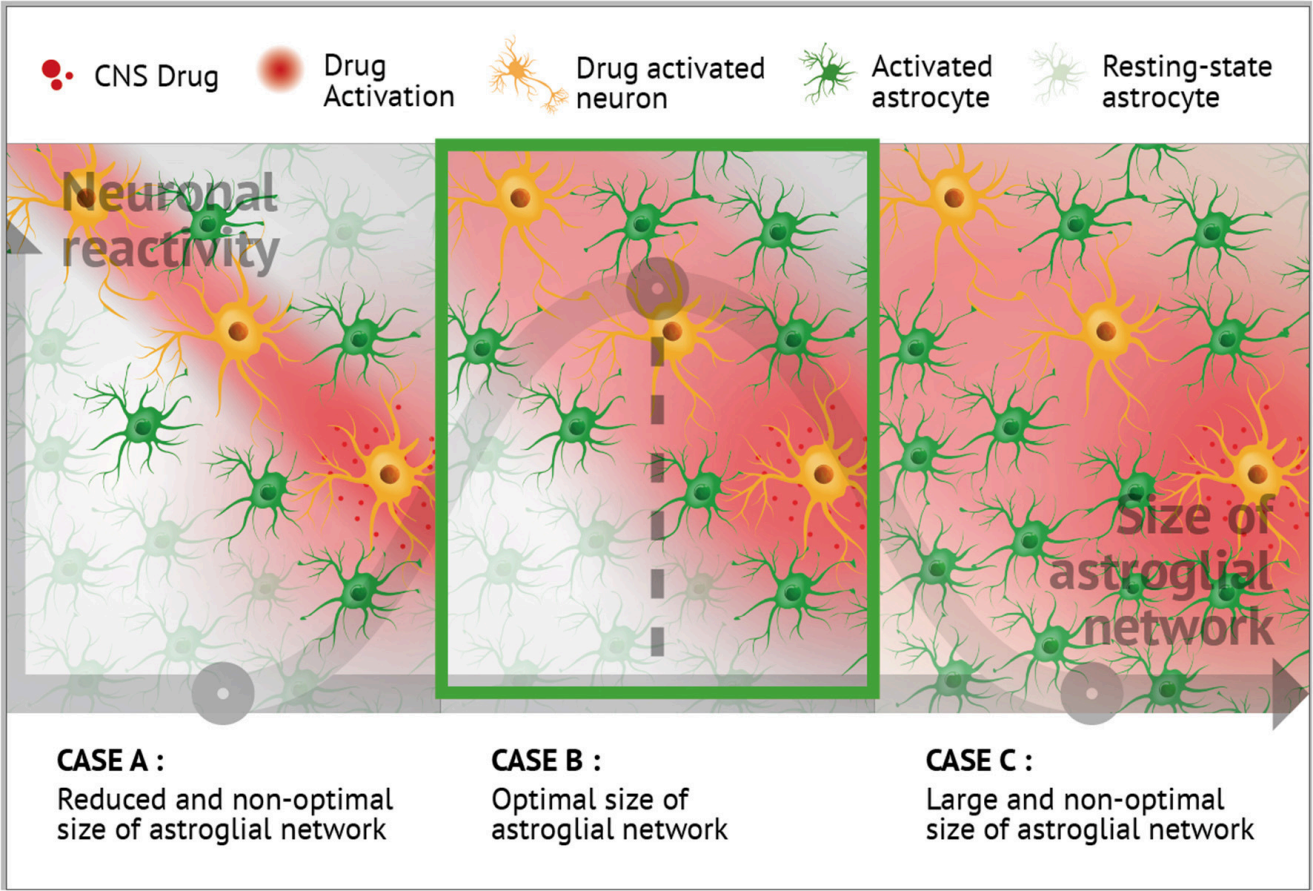
Putative role of astroglial connexins in the modulation of the CNS drug profiles. Neuronal and astrocyte networking activities are mutually regulated. Neuronal activity leads to an up-regulation of astrocyte Cx expression and channel function (Rouach et al., 2000, 2004, 2008; Koulakoff et al., 2008; Giaume et al., 2010; Pannasch et al., 2011, 2012, 2014; Roux et al., 2011). Interestingly, certain CNS-targeting drugs also appear to impact astrocyte Cxs (Giaume and Liu, 2012; Liu et al., 2013, 2016; Duchêne et al., 2016; Jeanson et al., 2016a,b). As presented in the three panels and based on literature, it can be hypothesized that optimal neuronal reactivity could be associated with an optimal size of local astroglial network **(B)**, as too large **(C)** or too reduced **(A)** syncytium might not adequately fuel metabolically active synapses during treatment with CNS drugs. Including aspects of drug action at the level of astrocytes, and more generally at the level of glial cells, opens up new avenues for potentially novel therapeutic applications.

Astrocytes are highly connected by Cx30 and Cx43, and this cell-cell communication strongly depends on neuronal activity. Conversely, neuronal processes are tightly tuned by astrocyte networks. The implications of neuroglial networking have furthermore been dissected in cognition, sleep-wake cycle, and sensory functions. Over the last decade, drugs that typically target neurons, such as antidepressant, antipsychotics, antiepileptic, psychostimulants, or anesthetics, have been shown to also modulate astroglial Cx expression and function. Additionally, reciprocal interactions, as well as mutual controls operate between neurons and Cx-formed astrocyte networks. Consequently, we envision that neuroglial networking may emerge as a new therapeutic target in neurological and psychiatric disorders. Two demonstrations of the interest in such targeting have been recently published in neuropathic pain (Jeanson et al., 2016a) and narcolepsy (Duchêne et al., 2016; Lu and Chen, 2016; see also Naus and Giaume, 2016). We hypothesized that astrocyte network size and structure should be adapted and optimized to neuronal demand, as a neither too large nor too reduced syncytium might adequately fuel metabolically active synapses, and this remains valid in the presence of CNS drugs (see Figure 4).

## Author contributions

All authors listed, have made substantial, direct and intellectual contribution to the work, and approved it for publication.

### Conflict of interest statement

MC is a co-founder and full-time employee of Theranexus. The other authors declare that the research was conducted in the absence of any commercial or financial relationships that could be construed as a potential conflict of interest.

## Abbreviations

CNS: Central Nervous System
Cx: Connexin
EAAT: Excitatory Amino Acid Transporter
ER: Endoplasmic Reticulum
gapFRAP: Gap-Fluorescence Recovery After Photobleaching
GJ: Gap Junction
GJC: gap junctional communication
GLUT1: Glucose Transporter type 1
GLUT3: Glucose Transporter type 3
HC: Hemichannel
IHC: Immunohistochemistry
IP_3_: Inositol trisphosphate
Kir1.4: Potassium inwardly-rectifying channel 1.4
LY: Lucifer Yellow
MCT1/4: Mono-Carboxylate Transporter 1/4
MCT2: Mono-Carboxylate Transporter 2.
